# A Novel COVID-19-Related Drug Discovery Approach Based on Non-Equidimensional Data Clustering

**DOI:** 10.3389/fphar.2022.813391

**Published:** 2022-02-21

**Authors:** Bolin Chen, Yourui Han, Xuequn Shang, Shenggui Zhang

**Affiliations:** ^1^ School of Computer Science, Northwestern Polytechnical University, Xi'an, China; ^2^ School of Mathematics and Statistics, Northwestern Polytechnical University, Xi'an, China; ^3^ Xi'an-Budapest Joint Research Center for Combinatorics, Northwestern Polytechnical University, Xi'an China

**Keywords:** COVID-19, matrix norm, Kronecker product, non-equidimensional data clustering, cluster center generating

## Abstract

The novel coronavirus disease (COVID-19) caused by severe acute respiratory syndrome coronavirus-2 (SARS-CoV-2) has spread all over the world. Since currently no effective antiviral treatment is available and those original inhibitors have no significant effect, the demand for the discovery of potential novel SARS-CoV-2 inhibitors has become more and more urgent. In view of the availability of the inhibitor-bound SARS-CoV-2 Mpro and PLpro crystal structure and a large amount of proteomics knowledge, we attempted using the existing coronavirus inhibitors to synthesize new ones, which combined the advantages of similar effective substructures for COVID-19 treatment. To achieve this, we first formulated this issue as a non-equidimensional inhibitor clustering and a following cluster center generating problem, where three essential challenges were carefully addressed, which are 1) how to define the distance between pairwise inhibitors with non-equidimensional molecular structure; 2) how to group inhibitors into clusters when the dimension is different; 3) how to generate the cluster center under this non-equidimensional condition. To be more specific, a novel matrix Kronecker product (*p*, *m*)-norm ${\left\|\cdot \right\|}_{p}^{m^{⊗}}$ was first defined to induce the distance *D*_*p*_(*A*, *B*) between two inhibitors. Then, the hierarchical clustering approach was conducted to find similar inhibitors, and a novel iterative algorithm–based Kronecker product (*p*, *m*)-norm was designed to generate individual cluster centers as the drug candidates. Numerical experiments showed that the proposed methods can find novel drug candidates efficiently for COVID-19, which has provided valuable predictions for further biological evaluations.

## 1 Introduction

Severe acute respiratory syndrome coronavirus-2 (SARS-CoV-2) has shockingly spread and caused huge social and economic destruction (Jin et al., 2020). SARS-CoV-2 has created an unprecedented health emergency around the world and till date 232,252,046 confirmed cases and 4,756,629 deaths have been documented. But no effective antiviral treatment is currently available, and new drugs are urgently needed (Ramesh et al., 2021).

Notably, SARS-CoV-2 is an envelope virus having a single-stranded positive-sense RNA genome (Ghosh et al., 2020; Elfiky and Azzam, 2021; Nejadi et al., 2021). In the replication and maturation stage of the virus, two polyproteins, i.e., pp1a and pp1ab, are promptly translated upon entry into the host cells. Then, two viral protease are the prerequisite enzymes of the viral replication and maturation which are raised upon proteolytic cleavage of pp1a and pp1b: one is main protease (Mpro) (Main protease Mpro also called chymotrypsin-like protease 3CLpro) and another is the papain-like protease (PLpro) enzymes (Lin et al., 2018; Ghosh et al., 2020; Elmezayen et al., 2021; Joshi et al., 2021). Both proteases are essential for SARS-CoV-2 viral replication and, thus, can be considered as drug-able targets (Ghosh et al., 2020).

On the one hand, Mpro and PLpro are progressing faster in molecular docking and target-based virtual screening research, and some progress has also been made in combinatorial chemistry and high-throughput screening of SARS-CoV-2 drugs. AL-Khafaji et al. (2021) use integrated computational approach to identify safe and rapid treatment for SARS-CoV-2. Das et al. (2021) have utilized a blind molecular docking approach to identify the possible inhibitors of the SARS-CoV-2 main protease. Enmozhi et al. (2021) evaluated the compound Andrographolide from *Andrographis paniculata* as a potential inhibitor of the main protease of SARS-COV-2 (Mpro) through *in silico* studies such as molecular docking, target analysis, toxicity prediction, and ADME prediction. On the other hand, screening through biological experiments is a time-consuming and energy-consuming event. Thus, there are also much works to accelerate in the search of inhibitors based on the chemical-informatics approach. Amin et al. (2021) did molecule identification and QSAR-based screening of in-house molecules active against putative SARS-CoV-2 PLpro. Ghosh et al. (2021) did QSAR-based screening of in-house molecules active against putative SARS-CoV-2 Mpro.

**FIGURE 1:**
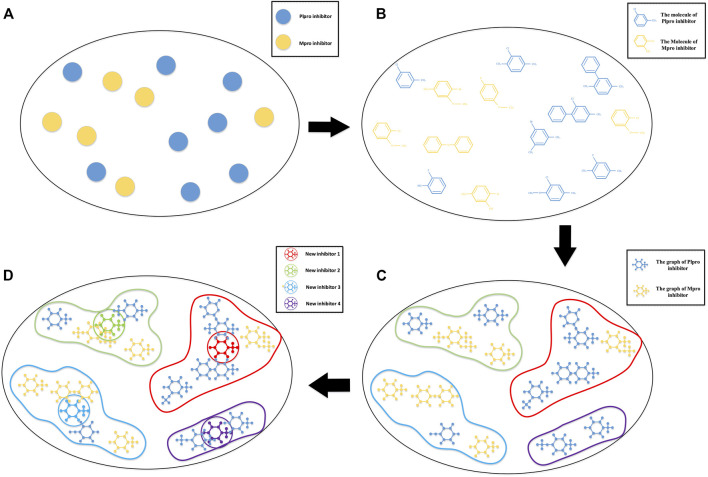
Schematic diagram of COVID-19 drug discovery. **(A)** A sketch map of main protease (Mpro) and active papain-like protease (PLpro) inhibitors with higher activity in a high-dimension space; **(B)** the molecular structure of these inhibitors in high-dimension space; **(C)** a graph representation of SARS-CoV PLpro and SARS-CoV Mpro inhibitors based on their three-dimensional molecular structure. The topological information of the inhibitor molecules is reserved, and the inhibitors are grouped by hierarchical clustering–based Kronecker product (*p*, *m*)-norm; **(D)** a novel iterative algorithm–based Kronecker product (*p*, *m*)-norm is used to generate the cluster centers of individual clusters.

These methods are more about screening original inhibitors or screening newly designed inhibitors. But designing new inhibitors from the biological level is a more tedious task. In view of the availability of the inhibitor-bound SARS-CoV-2 Mpro and PLpro crystal structure and a large amount of proteomics knowledge, we hope to use existing coronavirus inhibitors with similar structures to synthesize new inhibitors that have comprehensive advantages and may be effective against COVID-19. We model this as a non-equidimensional inhibitor clustering and the following cluster center generating problem. A schematic diagram of the idea is shown in Figure 1.

Although there are many methods to measure the similarity between different drugs, they are mainly based on the simplified molecular-input line-entry system (SMILES), ATC code, side effect, sequences, and GO of drug related targets (Huang et al., 2020). However, different inhibitors have different scales, i.e., some of them are large molecules, while others are small molecules, which makes it difficult to appropriately measure the full molecular structure of drugs. The graph representation of an inhibitor represent each atom as a vertex. Although it could contain the full structure information of the inhibitor, it also makes such representation result in different scales and dimensions for different inhibitors. In view of these, the novel drug discovery strategy needs to address the following three essential issues, which are 1) how to define the distance between pairwise inhibitors with different dimensions; 2) how to cluster inhibitors with different dimensions; and 3) how to generate the cluster center of similar inhibitors with different dimensions.

**FIGURE 2:**
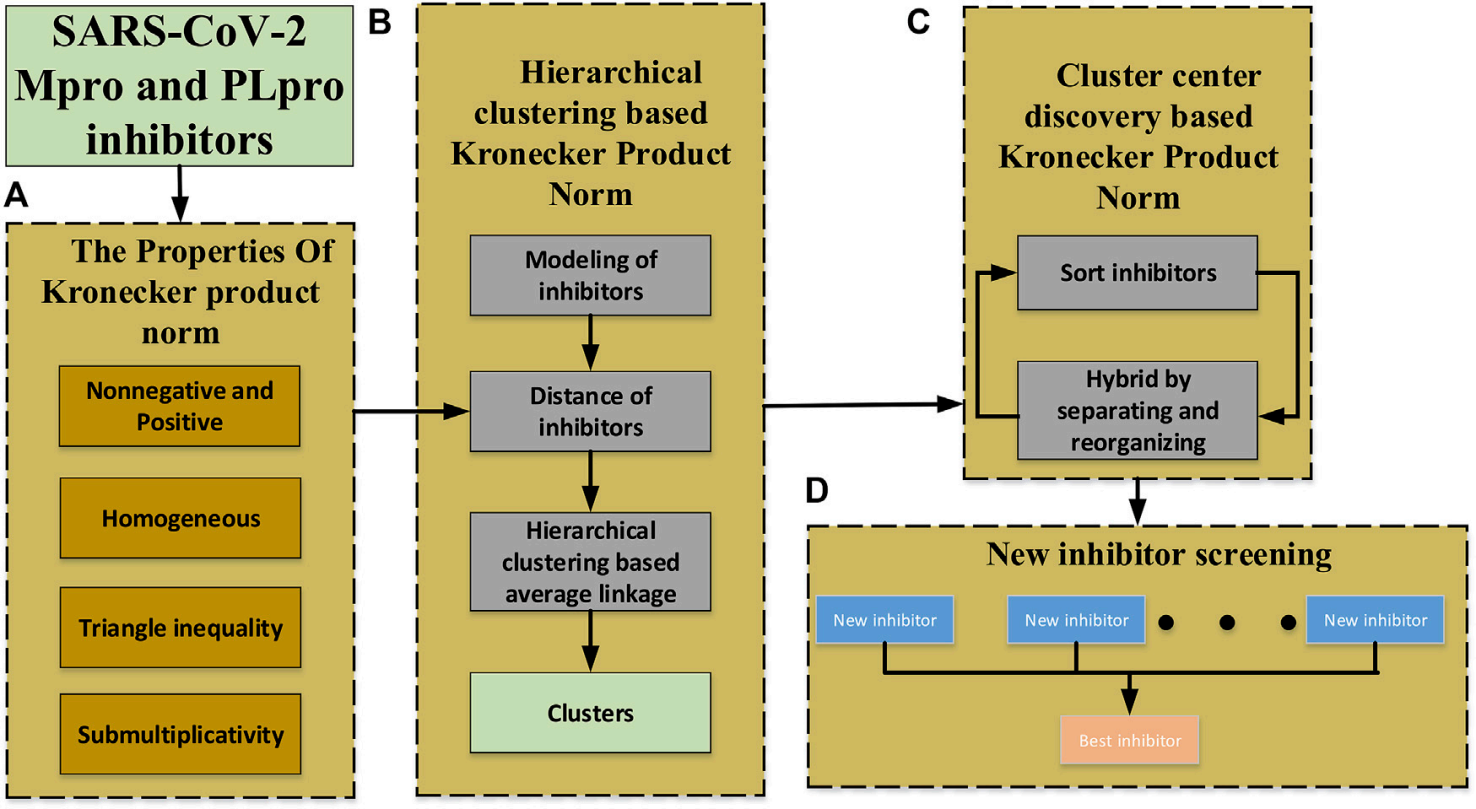
Schematic diagram of the proposed approach. **(A)** The definition of Kronecker product (*p*, *m*)-norm and its properties. **(B)** The clustering of non-equidimensional inhibitors based on Kronecker product (*p*, *m*)-norm and hierarchical clustering. **(C)** The cluster center generating based on the Kronecker product (*p*, *m*)-norm to get much newer inhibitors. **(D)** New inhibitors screening to find the best inhibitor.

To overcome these, we introduce a novel norm (matrix Kronecker product (*p*, *m*)-norm) ${\left\|\cdot \right\|}_{p}^{m^{⊗}}$ from the matrix norm to induce distance *D*_*p*_(*A*, *B*) between the inhibitors with different dimensions and propose a novel iterative algorithm–based Kronecker product (*p*, *m*)-norm to generate the cluster centers. A schematic diagram of the algorithm is shown in Figure 2.

## 2 Materials and Methods

### 2.1 Data Sources

We choose active main protease (Mpro) and active papain-like protease (PLpro) inhibitors whose pIC50 value are higher than the “activity threshold” as the “seed” set. Eventually, a total of 60 of them are selected, which are denoted as $\left\{s_{1},s_{2},\ldots ,s_{60}\right\}$ to be an example. (The active inhibitors are obtained from the articles of Amin et al., 2021 and Ghosh et al., 2021). The inhibitors' molecular structures are represented by SMILES and are shown in Table 1 (only a part of the inhibitors are displayed; all inhibitors' structures with SMILES notations are shown in the Supplementary Material).

**TABLE 1:** The simplified molecular-input line-entry system (SMILES) of main protease (Mpro) inhibitors and papain-like protease (PLpro) inhibitors.

Compound	SMILES notation
1-M	c1oc(cc1)C(=O)Oc1cncc(Br)c1
2-M	c1oc(cc1)C(=O)Oc1cncc(Cl)c1
3-M	c1cc(oc1C(=O)Oc1cncc(Cl)c1)c1ccc(Cl)cc1
4-M	c1c(sc2ccccc12)C(=O)Oc1cncc(Cl)c1
⋮	⋮

### 2.2 Distance of Inhibitors

There are many measures which can calculate the distance between pairwise inhibitors based their SMILES representation (Weininger, 1988). Most of them use descriptors to extract features and calculate the distance by using classic distance, such as, Manhattan distance, Euclidean distance, Chebyshev distance, and cosine distance. But the design of the descriptor in the feature extraction is not so easy, and it loses some of the information, which we are unsure is useful. On the one hand, some statistical characteristics, such as “SMILES atoms” *S*_*k*_, the combinations of two “SMILES atoms” *SS*_*k*_, and the combinations of three “SMILES atoms” *SSS*_*k*_, take into account the information on the lower-order neighbors of each atom in the molecule, such as the first-order neighbor, second-order neighbor, and third-order neighbor but lack information on the higher-order neighbors of the atom. Moreover, some can also define more optimal descriptors (Toropov et al., 2011), such as *BOND*, *NOSP*, and *HALO*. But those manually designed features only describe part of those inhibitor information, and some unknown important information may still be missed due to the complexity of the feature engineering.

**FIGURE 3:**
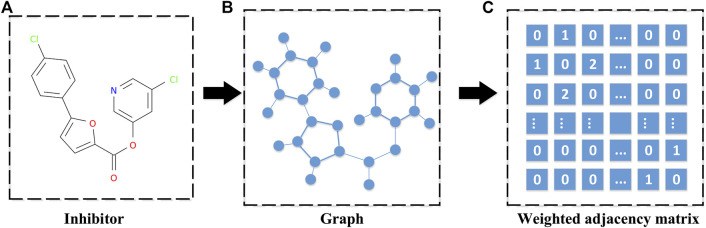
A diagram for weighted adjacency matrix of a given inhibitor. **(A)** The three-dimensional molecular structure of an inhibitor. **(B)** The corresponding graph of the inhibitor's molecular structure, which is obtained by treating the atoms in the molecular structure as nodes and the bonds in the molecular structure as edges. **(C)** The weighted adjacency matrix of the inhibitor's graph, and the weight determined by the type of chemical bond, where the single bond is 1 and the double bond is 2.

On the other hand, the SARS-CoV PLpro and SARS-CoV Mpro inhibitors can be represented as graphs through their three-dimensional molecular structure. These graphs contain all the topological information of the inhibitor molecules. For these graphs, some metrics, such as the count version of ECFP4 fingerprints, can be used to measure the distance between pairwise inhibitors with different dimensions by extracting features from the graphs. However, these features are not handy for generating new molecules without a structure yet from a set of similar inhibitors. Since we would like to synthesize a novel drug by recombining the structure of a set of highly related molecules, a novel matrix norm was proposed to measure the distance between the pairwise inhibitors with different dimensions without extracting their features. Hence, in this study, a graph representation is conducted to represent a given inhibitor by using its weighted adjacency matrix. The weight is determined by the type of the chemical bond, where the single bond is 1 and the double bond is 2. It is noted that different inhibitors may result in adjacency matrices with different sizes. A graph representation is shown in Figure 3.

#### 2.2.1 Kronecker Product Norm of Square Matrices

Traditionally, the distance between vectors can be induced by the norm of the vector, and the distance between matrices can be induced by the norm of the matrix. However, when the distance of two vectors (matrices) is induced by the currently known vector (matrix) norm, two vectors (matrices) are required to be of the same dimension. Therefore, it is an interesting problem whether a new norm can be defined to induce the distance between non-equidimensional vectors (matrices).

Considering that the matrix norm $\left\|A\right\|=\max\left\{\left\|A\cdot x\right\|:\left\|x\right\|=1\right\}$ is induced by the vector norm $\left\|x\right\|$ and that the Kronecker product ⊗ can increase the dimensionality of the matrix, we design a new function ${\left\|A\right\|}_{p}^{m^{⊗}}=\max\left\{{\left\|A⊗E_{m}\right\|}_{p}:{\left\|E_{m}\right\|}_{p}={\left\|I_{m}\right\|}_{p}=1\right\}$ , which is induced by matrix *p*-norm ${\left\|\cdot \right\|}_{p}$ on $R^{n\times n}$ . Next, we give proof that this new function ${\left\|A\right\|}_{p}^{m^{⊗}}$ is a matrix norm, such that we can use this novel matrix norm to induce its corresponding distance.

Theorem 1 The function ${\left\|A\right\|}_{p}^{m^{⊗}}=\max\left\{{\left\|A⊗E_{m}\right\|}_{p}:{\left\|E_{m}\right\|}_{p}={\left\|I_{m}\right\|}_{p}=1\right\}$ is a matrix norm on $R^{n\times n}$ and satisfies the following properties:

(i) ${\left\|A\right\|}_{p}^{m^{⊗}}\geq 0$ , unless A = 0, ${\left\|A\right\|}_{p}^{m^{⊗}}=0$ .
(ii) For any scalar α and any $A\in R^{n\times n}$ , ${\left\|\alpha A\right\|}_{p}^{m^{⊗}}=\left|\alpha \right|{\left\|A\right\|}_{p}^{m^{⊗}}$ .
(iii) For any two matrices $A\in R^{n\times n}$ and $B\in R^{n\times n}$ , ${\left\|A+B\right\|}_{p}^{m^{⊗}}\leq {\left\|A\right\|}_{p}^{m^{⊗}}+{\left\|B\right\|}_{p}^{m^{⊗}}$ .
(iv) For any two matrices $A\in R^{n\times n}$ and $B\in R^{n\times n}$ , ${\left\|AB\right\|}_{p}^{m^{⊗}}\leq {\left\|A\right\|}_{p}^{m^{⊗}}\cdot {\left\|B\right\|}_{p}^{m^{⊗}}$ .

Proof

(i) Nonnegative and positive:

$${\left\|A\right\|}_{p}^{m^{⊗}}=\text{max}\left\{{\left\|A⊗E_{m}\right\|}_{p}:{\left\|E_{m}\right\|}_{p}=1\right\}\geq 0,\;\text{unless}\;A=0,\;{\left\|A\right\|}_{p}^{m^{⊗}}=0.$$

(ii) Homogeneous:

$$\begin{array}{cc} {\left\|\alpha A\right\|}_{p}^{m^{⊗}} & =\text{max}\left\{{\left\|\alpha A⊗E_{m}\right\|}_{p}:{\left\|E_{m}\right\|}_{p}=1\right\}\\ & =\text{max}\left\{\left|\alpha \right|{\left\|A⊗E_{m}\right\|}_{p}:{\left\|E_{m}\right\|}_{p}=1\right\}\\ & =\left|\alpha \right|\cdot \text{max}\left\{{\left\|A⊗E_{m}\right\|}_{p}:{\left\|E_{m}\right\|}_{p}=1\right\}\\ & =\left|\alpha \right|\cdot {\left\|A\right\|}_{p}^{m^{⊗}}. \end{array}$$

(iii) Triangle inequality:

$$\begin{array}{cc} {\left\|A+B\right\|}_{p}^{m^{⊗}} & =\text{max}\left\{{\left\|\left(A+B\right)⊗E_{m}\right\|}_{p}:{\left\|E_{m}\right\|}_{p}=1\right\}\\ & \leq \text{max}\left\{{\left\|A⊗E_{m}\right\|}_{p}+{\left\|B⊗E_{m}\right\|}_{p}:{\left\|E_{m}\right\|}_{p}=1\right\}\\ & =\text{max}\left\{{\left\|A⊗E_{m}\right\|}_{p}:{\left\|E_{m}\right\|}_{p}=1\right\}+\text{max}\left\{{\left\|B⊗E_{m}\right\|}_{p}:{\left\|E_{m}\right\|}_{p}=1\right\}\\ & ={\left\|A\right\|}_{p}^{m^{⊗}}+{\left\|B\right\|}_{p}^{m^{⊗}} \end{array}$$

(iv) Submultiplicativity:

$$\begin{array}{cc} {\left\|AB\right\|}_{p}^{m^{⊗}} & =\text{max}\left\{{\left\|\left(A\cdot B\right)⊗E_{m}\right\|}_{p}:{\left\|E_{m}\right\|}_{p}=1\right\}\\ & =\text{max}\left\{{\left\|\left(A\cdot B\right)⊗\left(E_{m}\cdot I_{m}\right)\right\|}_{p}:{\left\|E_{m}\right\|}_{p}=1\right\}\\ & =\text{max}\left\{{\left\|\left(A⊗E_{m}\right)\cdot \left(B⊗I_{m}\right)\right\|}_{p}:{\left\|E_{m}\right\|}_{p}=1\right\}\\ & \leq \text{max}\left\{{\left\|A⊗E_{m}\right\|}_{p}\cdot {\left\|B⊗I_{m}\right\|}_{p}:{\left\|E_{m}\right\|}_{p}=1\right\}\\ & \leq \text{max}\left\{{\left\|A⊗E_{m}\right\|}_{p}:{\left\|E_{m}\right\|}_{p}=1\right\}\cdot \text{max}\left\{{\left\|B⊗I_{m}\right\|}_{p}:{\left\|E_{m}\right\|}_{p}=1\right\}\\ & \leq \text{max}\left\{{\left\|A⊗E_{m}\right\|}_{p}:{\left\|E_{m}\right\|}_{p}=1\right\}\cdot \text{max}\left\{{\left\|B⊗E_{m}\right\|}_{p}:{\left\|E_{m}\right\|}_{p}=1\right\}\\ & ={\left\|A\right\|}_{p}^{m^{⊗}}\cdot {\left\|B\right\|}_{p}^{m^{⊗}} \end{array}$$

Therefore, the function ${\left\|A\right\|}_{p}^{m^{⊗}}$ is a matrix norm, which is induced by the matrix *p*-norm ${\left\|A\right\|}_{p}$ , and it is also called the operator norm or least upper bound norm associated with the matrix *p*-norm ${\left\|A\right\|}_{p}$ . We name this novel matrix norm ${\left\|A\right\|}_{p}^{m^{⊗}}$ as the matrix Kronecker product (*p*, *m*)-norm.

#### 2.2.2 Distance of Different Dimension Square Matrices

The distance *D*_*p*_(*A*,*B*)^1^ of two inhibitors' weighted adjacency matrices with a different dimension is defined by the matrix Kronecker product (*p*, *m*)-norm ${\left\|\cdot \right\|}_{p}^{m^{⊗}}$ .

Definition 1 Let two matrices $A\in R^{n\times n}$ and $B\in R^{m\times m}$ , the distance *D*_*p*_(*A*,*B*)^1^ of matrices *A* and *B* is defined by

$$D_{p}{\left(A,B\right)}^{1}=\left\{\begin{array}{cc} {\left\|A-B\right\|}_{p}^{q^{⊗}}, & \text{if}\;\;n=m\\ \left|{\left\|A\right\|}_{p}^{{\left(q/n\right)}^{⊗}}-{\left\|B\right\|}_{p}^{{\left(q/m\right)}^{⊗}}\right|, & \text{if}\;\;n\neq m \end{array}\right.$$ (1)

where *q* is the least common multiple of *n* and *m*.

Meanwhile, we define the distance *D*_*p*_(*A*,*B*)^2^ of two inhibitors' square matrices with different scales by the idea of mapping *A* and *B* to the same dimension.

Definition 2 Let two matrices $A\in R^{n\times n}$ and $B\in R^{m\times m}$ , the distance *D*_*p*_(*A*,*B*)^2^ of matrices *A* and *B* is defined by

$$D_{p}{\left(A,B\right)}^{2}={\left\|A⊗I_{q/n}-B⊗I_{q/m}\right\|}_{p},$$ (2)

where *q* is the least common multiple of *n* and *m*.

### 2.3 Hierarchical Clustering–Based Kronecker Product Norm

Once the pairwise distances between any two inhibitors are obtained by *D*_*p*_(*A*,*B*)^*i*^, *i* ∈ {1, 2}, a clustering procedure can be conducted to group similar inhibitors, where the shorter the distance between two inhibitors, the higher the possibility that they are grouped into the same cluster. However, not every clustering method works in this case of non-equidimensional data clustering. If the dimensions of two inhibitors are the same, then the cluster center can be naturally obtained in an averagely weighted manner. But if the dimensions of two or more weighted adjacency matrices are different, the center of a group of inhibitors is unavailable by using the above averagely weighted manner. This means, we cannot use the clustering methods that are based on the centroid linkage or rely on the cluster center, such as k-means. In this study, the hierarchical clustering method that D'Andrade (1978) based on the average linkage of two clusters

$$d_{avg}\left(C_{i},C_{j}\right)=\frac{1}{\left|C_{i}|*|C_{j}\right|}\underset{x\in C_{i}}{\sum }\underset{z\in C_{j}}{\sum }dist\left(x,z\right).$$

was employed to test our proposed method.

### 2.4 Cluster Center Discovery–Based Kronecker Product Norm

After clustering, a list of clusters $\left\{C_{1},C_{2},\ldots ,C_{m}\right\}$ can be obtained, and the inhibitors in the same cluster $C_{i}=\left\{s_{i}^{1},s_{i}^{2},\ldots ,s_{i}^{n}\right\}$ can be hybridized to generated new predictions by iteratively separating and reorganizing.

We use a “bottom–up” aggregation strategy to design an iterative algorithm with the heuristic measure function *f*, which is constructed by *D*_*p*_(*A*,*B*)^*i*^. First, for cluster $C=\left\{s_{1},s_{2},\ldots ,s_{n}\right\}$ , $C\in \left\{C_{1},C_{2},\ldots ,C_{m}\right\}$ , each inhibitor *s*_*i*_ is regarded as an initial sample, and then the two closest samples $s_{j^{*}}$ and $s_{k^{*}}$ are found and merged in each step of the algorithm operation.

**FIGURE 4:**
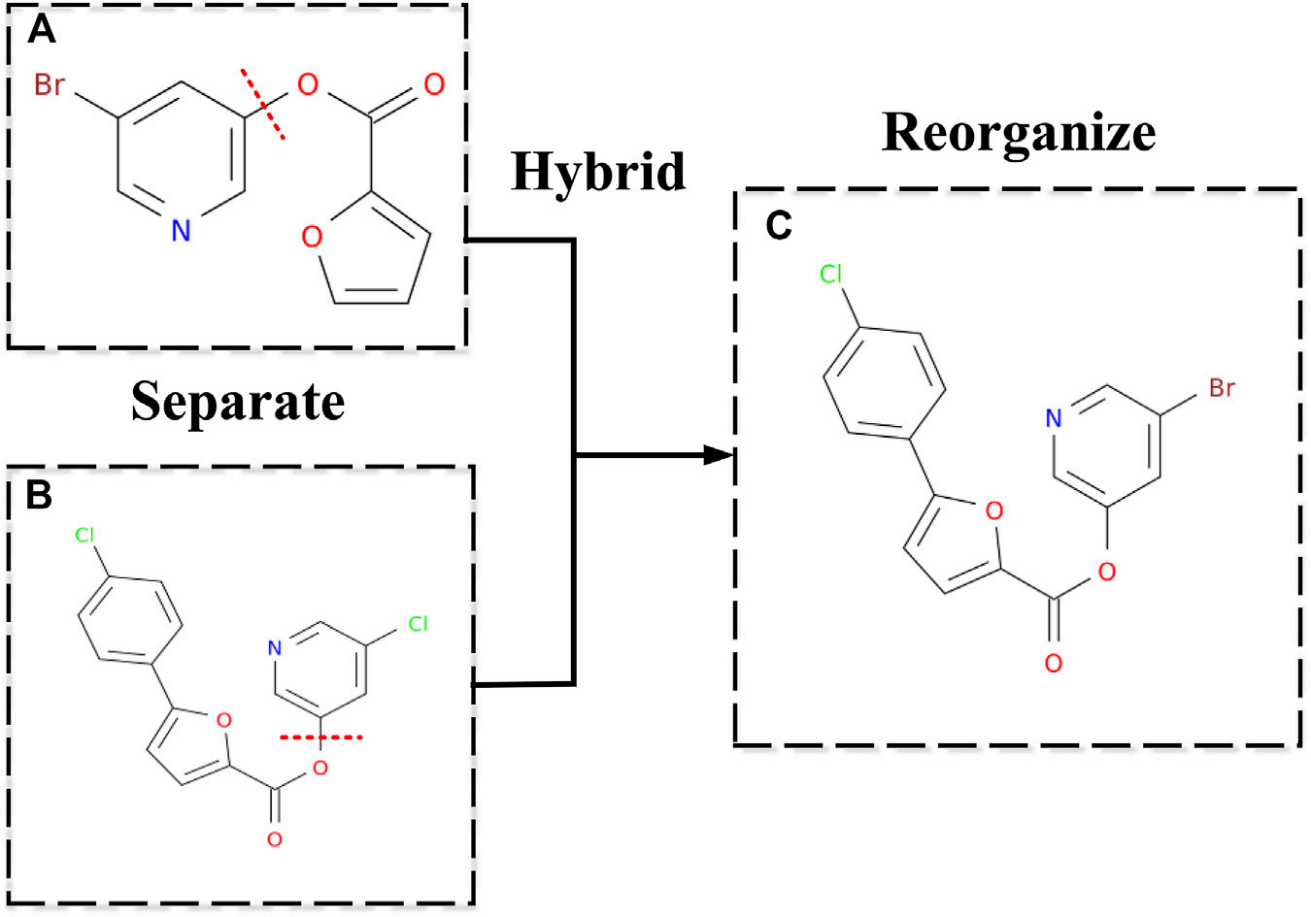
New inhibitor's molecular structure by hybridizing. **(A,B)** Two inhibitors were employed as examples to show how a new drug can be generated. Separating means randomly disconnecting a cut edge in the two inhibitor molecules' graphs, which will divide the two inhibitor molecules into four parts. **(C)** Reorganizing means randomly choosing two of the four parts, which do not belong to the same inhibitor molecule, to combine the two parts and make it a new molecule by adding an edge.

Then through merging to the hybrid, the closest two inhibitors $s_{j^{*}}$ and $s_{k^{*}}$ in the same cluster by separating and reorganizing, we will get much newer inhibitors *s*′. Separating means randomly disconnecting a cut edge in the two inhibitor molecules' graphs, which will divide the two inhibitor molecules into four parts. Reorganizing means randomly choosing two of the four parts which do not belong to the same inhibitor molecule, to combine the two parts and making it a new molecule by adding an edge. The schematic diagram is shown in Figure 4.

Finally, an intermediate product *s*∗ will be chosen by the heuristic measure function $f\left(s^{\prime }\right)=g\left(s_{j^{*}}\right)*D_{p}{\left(s_{j^{*}},s^{\prime }\right)}^{i}+g\left(s_{k^{*}}\right)*D_{p}{\left(s_{k^{*}},s^{\prime }\right)}^{i}=w_{j^{*}}*D_{p}{\left(s_{j^{*}},s^{\prime }\right)}^{i}+w_{k^{*}}*D_{p}{\left(s_{k^{*}},s^{\prime }\right)}^{i}$ from new inhibitors *s*′ and taken in the place of these two inhibitors.

But the intermediate product *s*∗ is not the original two inhibitors after all, we therefore set a weight for each inhibitor $W=\left\{w_{i}|w_{i}=1,i=1,2,\ldots ,n\right\}$ , and as the number of hybridizations increases, the weight of the corresponding inhibitors will be larger. In this way, when calculating the distance, the inhibitors with more hybridization will have a greater distance than before.

With the iteration of the algorithm, the cluster set will remove the original two inhibitors and add an intermediate product until there is only one inhibitor left in the cluster set. This inhibitor is approximately the cluster center of the cluster set. The pseudo code of the proposed algorithm is described as follows. The code is available and can be downloaded from the Internet at https://www.github.com/HenryHan1997/drug_discover.

## 3 Experiments and Results

### 3.1 The Clustering of Inhibitors

We get the weighted adjacency matrix from the active main protease (Mpro) and papain-like protease (PLpro) inhibitors' structure. Then, we use *D*_2_(*A*,*B*)^2^ as the distance between the pairwise inhibitors and use the average linkage *d*_*avg*_(*C*_*i*_, *C*_*j*_) as the distance between the two clusters to cluster the “seed” set by AGNES hierarchical clustering (Kaufman and Rousseeuw, 2009).

**FIGURE 5:**
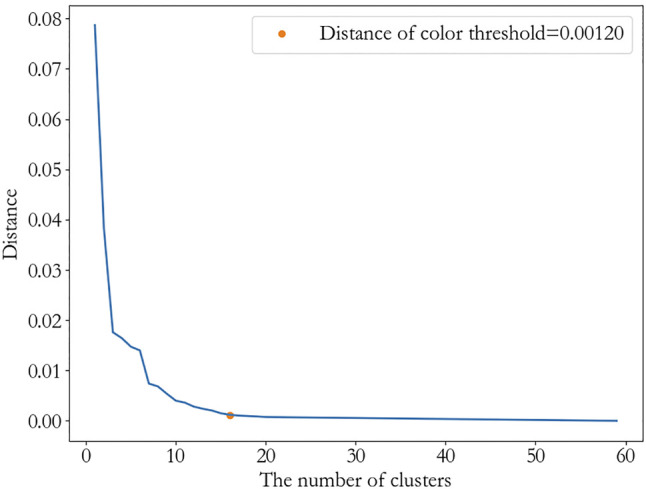
The color threshold corresponding to the different numbers of clusters.

**FIGURE 6:**
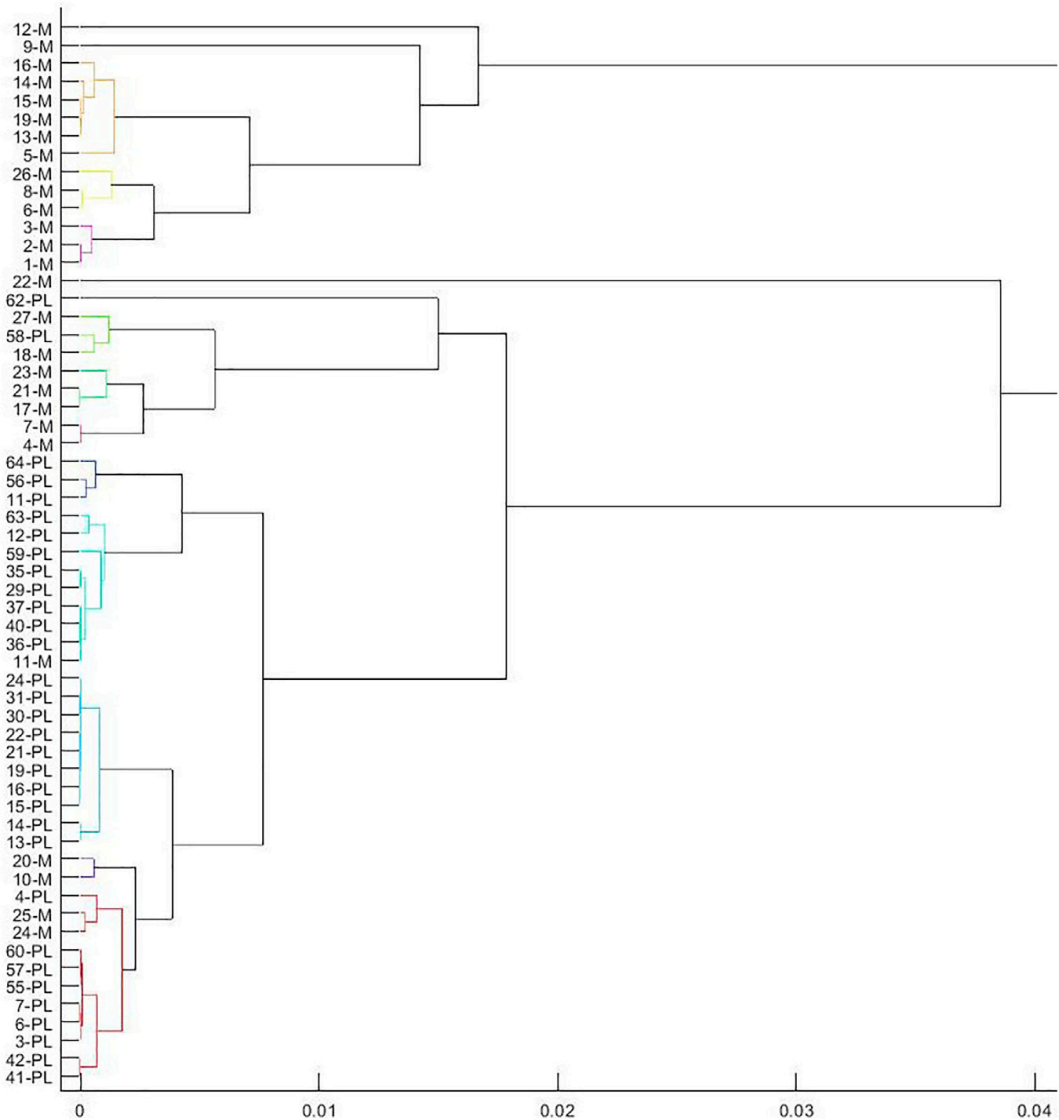
Clusters obtained from the hierarchical clustering tree by using the average linkage.

Then, we get a tree-like hierarchical structure of the inhibitors according to the average linkage. The threshold is chosen as 0.0012, since it is the elbow position according to the Figure 5. This indicates the distance between the clusters is as large as possible, while the distance within the clusters is as small as possible. After removing clusters less than two inhibitors, 10 clusters are obtained, which are $\left\{C_{1},C_{2},\ldots ,C_{10}\right\}$ . They are marked with different colors in Figure 6.

From the results, it can be seen that basically the inhibitors of the same type are still in the same cluster after clustering, i.e., papain-like protease inhibitors 11 − *PL*, 56 − *PL*, 64 − *PL* are in the same cluster, and the main protease inhibitors 5 − *M*, 13 − *M*, 14 − *M*, 15 − *M*, 16 − *M*, 19 − *M* are in the same cluster. This shows that our proposed distance *D*_*p*_(*A*,*B*)^*i*^ based on the Kronecker product (*p*, *m*)-norm ${\left\|\cdot \right\|}_{p}^{m^{⊗}}$ can indeed measure the similarity between pairwise inhibitors of different dimensions.

### 3.2 The Cluster Centers of Inhibitors

We chose one cluster, which contains papain-like protease inhibitors 11 − *PL*, 56 − *PL*, 64 − *PL* as an example and used **Algorithm 1** with *p* = 2 and *D*_2_(*A*,*B*)^2^ to discover new inhibitors and count the number of occurrences. Finally, we selected the three most frequent occurrences for analysis, which are shown in Table 2. The new inhibitors are considered to be valid because their SMILES representation can be successfully parsed by the RDKit.

**TABLE 2:** The three most frequently appeared inhibitors.

New inhibitor	SMILES notation
First	CN[C@H](COC)c1ccccc1
Second	COCC
Third	c1c(N)cccc1

SMILES, simplified molecular-input line-entry system.

Algorithm 1 Cluster center generation.

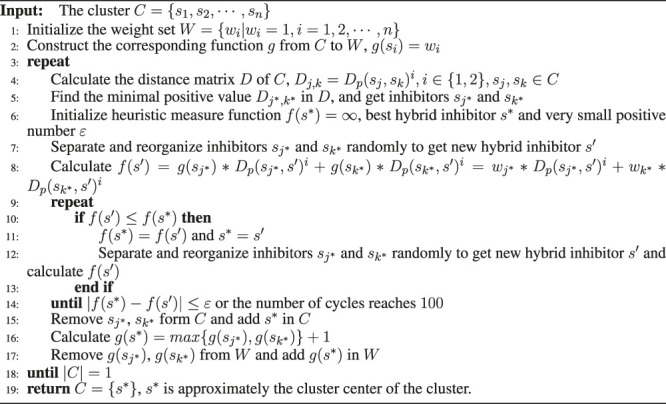

**FIGURE 7:**
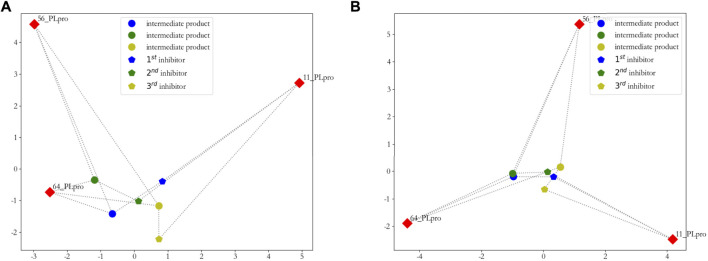
Two-dimensional visualization of inhibitors. **(A)** principal component analysis (PCA) is used to reduce the dimensionality of the distance matrix, which is calculated from these three new inhibitors, intermediate products, and original seeds; **(B)** sparse principal component analysis (SPCA) is used to reduce the dimensionality of the distance matrix, which is calculated from these three new inhibitors, intermediate products, and original seeds.

To show that our discovered new inhibitors are approximately the cluster centers, we visualized them in a two-dimensional plane. We used principal component analysis (PCA) and sparse PCA to reduce the dimensionality of the distance matrix, which is calculated from these three new inhibitors, intermediate products, and the original seeds by *D*_2_(*A*,*B*)^2^. The results are shown in Figure 7.

**FIGURE 8:**
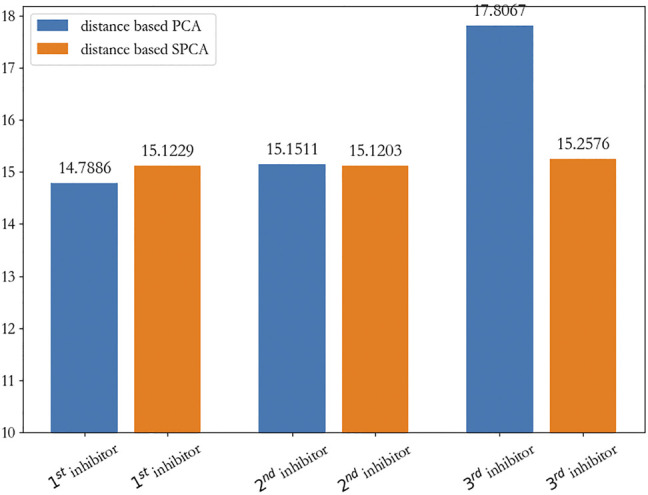
The sum of the distance from the new inhibitor to the seeds.

From Figure 7A, we can clearly see that the first and second new inhibitors are probably in the center of the cluster, and the third new inhibitor does not perform well; from Figure 7B, it is evident that the three new inhibitors are probably in the center of the cluster. On the whole, we calculated the sum of the distance from the new inhibitor to the seeds, and showed that the first new inhibitor performs best, which is shown in Figure 8.

Finally, we calculated the quantitative estimate of drug-likeness (QED) of the new inhibitors and original inhibitors, which is synthesized by using eight descriptors. The descriptors contain *MW*, *logP*, *HBA*, *HBD*, *PSA*, *ROTB*, *AROM*, and *ALERTS* (Brown et al., 2019). The QED of the first new inhibitor reached 0.731, which is the highest and is higher than the original three inhibitors (11 − *PL*, 56 − *PL*, 64 − *PL*). The results are shown in Table 3.

**TABLE 3:** Some properties of new inhibitors and original inhibitors.

Inhibitors	*MW*	*logP*	*HBA*	*HBD*	*PSA*	*ROTB*	*AROM*	*ALERTS*	*QED*
11 − *PL*	416.56	5.12	3	1	41.57	7	3	0	0.581
56 − *PL*	334.42	4.06	3	2	64.35	5	3	1	0.692
64 − *PL*	294.35	4.25	3	0	47.28	0	2	1	0.682
First	165.24	1.59	2	1	21.26	4	1	0	**0.731**
Second	60.10	0.65	1	0	9.23	1	0	0	0.432
Third	93.13	1.27	1	1	26.02	0	1	1	0.480

The bold values indicate the best performer in that column. The values of this column are the weighted combination of the previous columns, this is the reason why only the best value of this column is bold.

**FIGURE 9:**
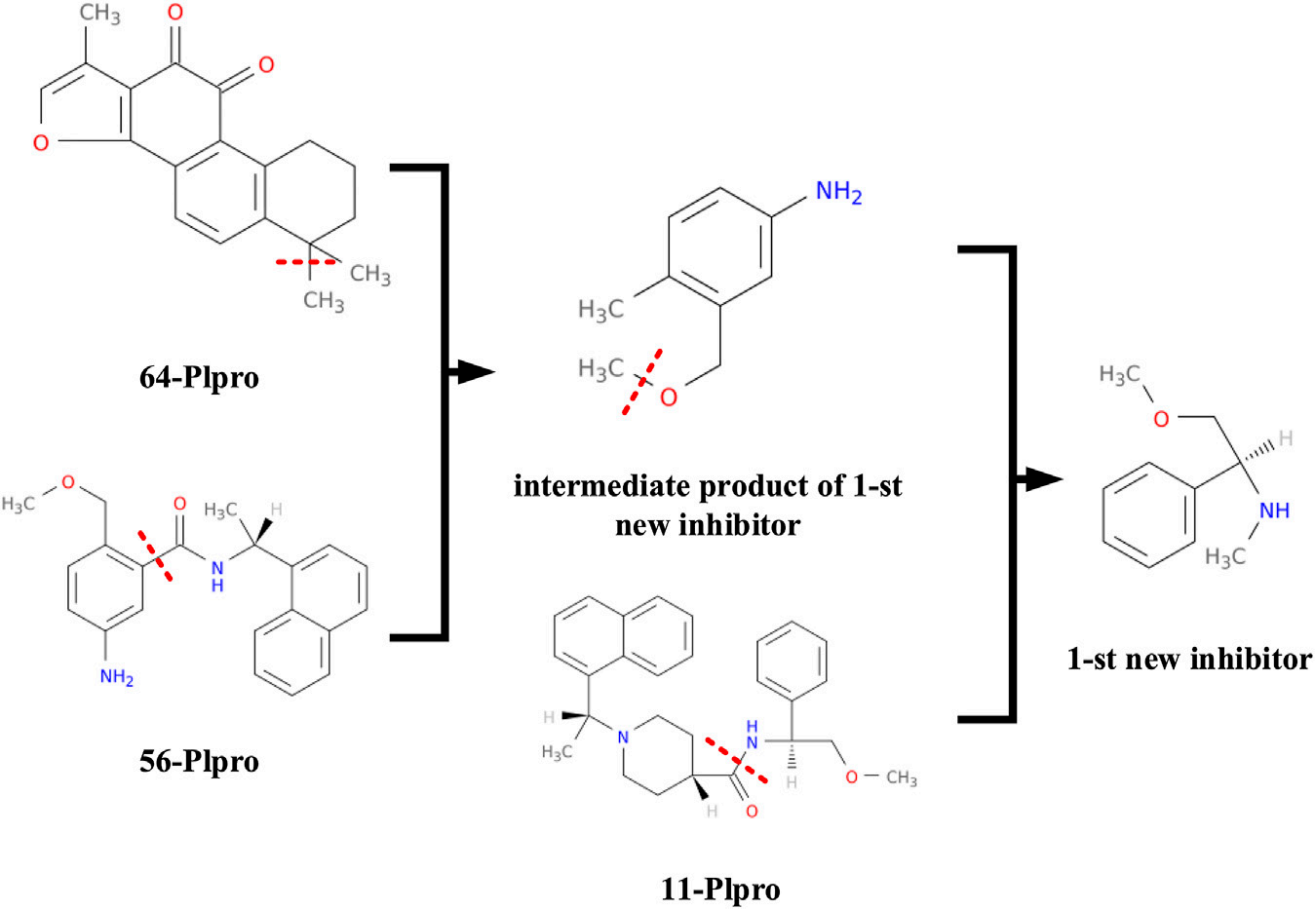
The first inhibitor's synthetic route.

At the same time, we also record the synthetic route of the first new inhibitor for analysis, which is shown in Figure 9. The first new inhibitor is obtained by recombining papain-like protease inhibitors 56 − *PL* and 64 − *PL* to form an intermediate product *c*1(*c*(*ccc*(*c*1)*N*)*C*)*COC*, and then separating and combining the intermediate product *c*1(*c*(*ccc*(*c*1)*N*)*C*)*COC* and papain-like protease inhibitor 11 − *PL*.

This kind of procedure is not possible by using the SMILE–based method directly. But using the proposed graph representation, we can easily generate more number of potential new drugs by combining information of currently known related drugs.

## 4 Conclusion and Future Research Directions

In view of the availability of the inhibitor-bound SARS-CoV-2 Mpro and PLpro crystal structure and a large amount of proteomics knowledge, we hope to synthesize inhibitors with similar structures or functions to discover a new inhibitor which may has comprehensive advantages. We model it as the discovery problem of the cluster center and propose a novel approach to discover some new inhibitors by finding cluster centers of known coronavirus inhibitors, such as SARS-CoV PLpro and SARS-CoV Mpro inhibitors.

Considering the inhibitors' different dimensions and that that alignment-free methods may lose some important information in feature engineering, we induce a novel norm (matrix Kronecker product (*p*, *m*)-norm) ${\left\|\cdot \right\|}_{p}^{m^{⊗}}$ from the matrix norm to define the distance *D*_*p*_(*A*,*B*)^*i*^ of inhibitors with different dimensions. Converting the three-dimensional structure of the inhibitor into a graph, and obtaining the corresponding two-dimensional matrix representation, we then measure the distance by *D*_*p*_(*A*,*B*)^*i*^. This approach preserves the inhibitors' information as much as possible, such that we can perform clustering to obtain those inhibitors with similar structures or functions. Meanwhile, we propose cluster center generation algorithm **Algorithm 1** to approximate the cluster centers by separating and reorganizing the inhibitors. In this way, we can easily obtain some new inhibitors for subsequent screening, which may have comprehensive advantages from the active inhibitors.

Also, this method has some drawbacks and limitations that require us to further consider and explore. The current method does not consider the side effects of inhibitors, and we should consider this matter when merging to hybridize the old inhibitors, such that the new inhibitors are excellent.

## Data Availability

The original contributions presented in the study are included in the article/Supplementary Material, and further inquiries can be directed to the corresponding authors.
