# Supplementary material for: A Novel COVID-19-Related Drug Discovery Approach Based on Non-Equidimensional Data Clustering
Source: Front Pharmacol. 2022 Feb 21;13:813391. doi: 10.3389/fphar.2022.813391 (PMC8900916; doi:10.3389/fphar.2022.813391)
Supplement: Supplementary file 1 [file DataSheet1.PDF]

Supplementary Material

1 SUPPLEMENTARY DATA

We choose a total of 60 active main protease (Mpro) and active papain-like protease (PLpro) inhibitors, whose pIC_{50} value higher than the 'activity threshold', as "seed" set $\{s_1, s_2, \cdots, s_{60}\}$ to be an example (The active inhibitors are obtained from Amin's paper (Amin et al., 2021) and Ghosh's paper (Ghosh et al., 2021)). The inhibitors' molecular are represented by simplified molecular input line entry system (SMILES), which are shown in Table.S1,S2.

Table S1. The SMILES of main protease (Mpro) inhibitors

Compound	SMILES notation
1-M	c1oc(cc1)C(=O)Oc1cncc(Br)c1
2-M	c1oc(cc1)C(=O)Oc1cncc(Cl)c1
3-M	c1cc(oc1C(=O)Oc1cncc(Cl)c1)c1ccc(Cl)cc1
4-M	c1c(sc2ccccc12)C(=O)Oc1cncc(Cl)c1
5-M	c1c([nH]c2ccccc12)C(=O)Oc1cncc(Cl)c1
6-M	c1c(sc2ccccc12)C(=O)Oc1cncc(Cl)c1
7-M	CC(C)C(NC(=O)C(NC(=O)C)C(C)O)C(=O)<br>NC(CC1CCCCC1)C(=O)NC(Cc1cnc[nH]1)C=O
8-M	c1c(oc2ccccc12)C(=O)Oc1cncc(Cl)c1
9-M	c1nc(cs1)C(=O)Oc1cncc(Cl)c1
10-M	c1c(Cl)c(cc(c1Cl)S(=O)(=O)c1c(cc(<br>cc1N(=O)=O)C(F)(F)F)N(=O)=O)C
11-M	c1cc(OC)cc(c1)C(=O)Oc1cncc(Cl)c1
12-M	c1cc(ccc1OC(=O)C(=C(Cl)Cl)Cl)S(=O)<br>(=O)c1ccc(OC(=O)C(=C(Cl)Cl)Cl)cc1
13-M	c1c(sc2ccccc12)CN1C(=O)C(=O)c2cc(I)ccc12
14-M	c1c(sc2ccccc12)CN1C(=O)C(=O)c2cccc(Br)c12
15-M	c1c(sc2ccccc12)CN1C(=O)C(=O)c2cccc(c12)N(=O)=O
16-M	c1cc(oc1C#Cc1ccccc1)C(=O)Sc1nnc([nH]1)C(F)(F)F
17-M	CC(C)CC(NC(=O)C(NC(=O)C(C)NC(=O)OCc1c<br>cccc1)C(C)C)C(=O)NC(CCC(=O)N)C=N
18-M	CC(C)(C)NC(=O)C(N(C(=O)c1ccco1)c1ccc(cc1)C(C)(C)C)c1cccnc1
19-M	c1c(sc2ccccc12)CN1C(=O)C(=O)c2cc(F)ccc12
20-M	N(C(=O)c1sc(cc1)c1cc(nn1C)C(F)(F)F)c1onc(c1N(=O)=O)C
21-M	CC(C)C[C@H](NC(=O)[C@H](NC(=O)[C@@H](C)NC(=O)<br>[C@@H](CO)NC(=O)C)C(C)C)C(=O)N[C@@H](Cc1cnc[nH]1)C=O
22-M	Nc1ncc(c(n1)N)S(=O)(=O)c1ccc(Cl)cc1
23-M	CCC(C)(C)NC(=O)[C@@H](N(C(=O)Cn1nnc2ccccc12)<br>c1ccc(NC(=O)C)cc1)c1cccn1C
24-M	c1cc(C#N)cc2C(=O)C(=O)N(c12)Cc1c(noc1C)C
25-M	c1cc(c2ccc3C4=C(C(=O)C(=O)c3c2c1)C(C)CO4)C
26-M	c1cc(I)cc2C(=O)C(=O)N(c12)Cc1ccc(F)cc1Cl
27-M	N(c1cc(n[nH]1)c1cc(oc1C)C(C)(C)C)S(=O)(=O)c1sccc1

Table S2. The SMILES of papain-like protease (PLpro) inhibitors

Compound	SMILES notation
3-PL	c1(ccccc1C)C(=O)N[C@@H](c1c2c(ccc1)cccc2)C
4-PL	c1(cc(ccc1C)NC(=O)C)C(=O)N[C@@H](c1c2c(ccc1)cccc2)C
6-PL	c1(cc(ccc1C)N)C(=O)N[C@@H](c1c2c(ccc1)cccc2)C
7-PL	[N@@H+]1(CC[C@@H](CC1)C(=O)NCc1ccccc1)[C@H](C)c1cccc2ccccc12
11-PL	N1(CC[C@@H](CC1)C(=O)N[C@H](COC)c1ccccc1)[C@H](C)c1cccc2ccccc12
12-PL	[N@H+]1(CC[C@@H](CC1)C(=O)NCc1ccc(cc1)CC)[C@H](C)c1cccc2ccccc12
13-PL	[N@@H+]1(CC[C@@H](CC1)C(=O)NCc1ccc(cc1)C(=O)NC)[C@H](C)c1cccc2ccccc12
14-PL	[N@@H+]1(CC[C@@H](CC1)C(=O)NCc1cc(ccc1)C(=O)NC)[C@H](C)c1cccc2ccccc12
15-PL	[N@@H+]1(CC[C@@H](CC1)C(=O)NCc1ccc(cc1)NC(=O)C)[C@H](C)c1cccc2ccccc12
16-PL	[N@@H+]1(CC[C@@H](CC1)C(=O)NCc1cc(ccc1)NC(=O)C)[C@H](C)c1cccc2ccccc12
19-PL	[N@@H+]1(CC[C@@H](CC1)C(=O)NCc1ccc(cc1)Cl)[C@H](C)c1cccc2ccccc12
21-PL	[N@@H+]1(CC[C@@H](CC1)C(=O)NCc1ccc(cc1)F)[C@H](C)c1cccc2ccccc12
22-PL	[N@@H+]1(CC[C@@H](CC1)C(=O)NCc1cc(ccc1)F)[C@H](C)c1cccc2ccccc12
24-PL	[N@@H+]1(CC[C@@H](CC1)C(=O)NCc1cc(ccc1)F)[C@@H](C)c1c2cccnc2ccc1
29-PL	[N@@H+]1(CC[C@H](CC1)C(=O)NCc1cc(ncc1)OC)[C@H](C)c1cccc2ccccc12
30-PL	[N@H+]1(CC[C@@H](CC1)C(=O)NCCc1ccc(cc1)Cl)[C@H](C)c1cccc2ccccc12
31-PL	[N@H+]1(CC[C@@H](CC1)C(=O)NCCc1cc(ccc1)F)[C@H](C)c1cccc2ccccc12
35-PL	N1(CC[C@@H](CC1)C(=O)NCc1c(cccc1)OC)[C@H](C)c1cccc2ccccc12
36-PL	N1(CC[C@@H](CC1)C(=O)NCc1cc(ccc1)OC)[C@H](C)c1cccc2ccccc12
37-PL	N1(CC[C@@H](CC1)C(=O)NCc1ccc(cc1)OC)[C@H](C)c1cccc2ccccc12
40-PL	N1(CC[C@H](CC1)C(=O)NCc1cc(ccc1)OC)[C@@H](C)c1cc2c(cc1)cccc2
41-PL	N1(CC[C@@H](CC1)C(=O)NCc1cc2c(cc1)OCO2)[C@H](C)c1cccc2ccccc12
42-PL	N1(CC[C@H](CC1)C(=O)NCc1cc2c(cc1)OCO2)[C@@H](C)c1cccc2ccccc12
55-PL	C(=O)(c1c(ccc(c1)C#N)C)N[C@H](C)c1cccc2ccccc12
56-PL	C(=O)(c1c(ccc(c1)N)COC)N[C@H](C)c1cccc2ccccc12
57-PL	C(=O)(c1c(ccc(c1)I)C)N[C@H](C)c1cccc2ccccc12
58-PL	C(=O)(c1c(ccc(c1)CNC(=O)OC(C)(C)C)C)N[C@H](C)c1cccc2ccccc12
59-PL	C(=O)(c1c(ccc(c1)CNC)C)N[C@H](C)c1cccc2ccccc12
60-PL	C(=O)(c1c(ccc(c1)CN)C)N[C@H](C)c1cccc2ccccc12
62-PL	N1=C([C@@H]2[C@@H]([NH+]=C1N)N=C[NH2+]2)S
63-PL	C1=CC(=O)N(C1=O)CC
64-PL	C1CC(c2c(C1)c1c(cc2)c2c(C(=O)C1=O)c(co2)C)(C)C

REFERENCES

Amin, S. A., Ghosh, K., Gayen, S., and Jha, T. (2021). Chemical-informatics approach to covid-19 drug discovery: monte carlo based qsar, virtual screening and molecular docking study of some in-house molecules as papain-like protease (plpro) inhibitors. *Journal of Biomolecular Structure and Dynamic* 39, 4764–4773. doi:10.1080/07391102.2020.1780946

Ghosh, K., Amin, S. A., Gayen, S., and Jha, T. (2021). Chemical-informatics approach to covid-19 drug discovery: exploration of important fragments and data mining based prediction of some hits from natural origins as main protease (mpro) inhibitors-sciencedirect. *Journal of Molecular Structure* 1224, 129026. doi:10.1016/j.molstruc.2020.129026
